# Adjuvant treatment of resectable biliary tract cancer with cisplatin plus gemcitabine: A prospective single center phase II study

**DOI:** 10.1186/s12885-017-3967-0

**Published:** 2018-01-11

**Authors:** Alexander R. Siebenhüner, Heike Seifert, Helga Bachmann, Burkhardt Seifert, Thomas Winder, Jonas Feilchenfeldt, Stefan Breitenstein, Pierre-Alain Clavien, Roger Stupp, Alexander Knuth, Bernhard Pestalozzi, Panagiotis Samaras

**Affiliations:** 1 Department of Medical Oncology, University Hospital Zurich, Rämistrasse 100, 8091 Zurich, CH Switzerland; 2 Swiss HBP Center, University Hospital Zurich, Rämistrasse 100, 8091 Zurich, CH Switzerland; 3 Epidemiology, Biostatistics and Prevention Institute, University of Zurich, Hirschengraben 84, 8001 Zurich, CH Switzerland; 4 National Center for Cancer Care and Research, Doha, Qatar; 5 Department of Surgery, Cantonal Hospital of Winterthur, Brauerstrasse 15, 8401 Winterthur, Switzerland

**Keywords:** Adjuvant chemotherapy, Biliary tract cancer, Cholangiocellular carcinoma, Gallbladder cancer, Cisplatin and gemcitabine, Feasibility

## Abstract

**Background:**

Biliary tract cancer (BTC) is a dismal disease, even after curative intent surgery. We conducted this prospective, non-randomized phase II study to evaluate the feasibility and efficacy of cisplatin and gemcitabine as adjuvant treatment in patients with resected BTC.

**Methods:**

Patients initially received gemcitabine 1000 mg/m^2^ alone on days 1, 8 and 15 every 28-days for a total of six cycles (single agent cohort), and after protocol amendment a combination therapy with gemcitabine 1000 mg/m^2^ and cisplatin 25 mg/m^2^ on days 1 and 8 was administered every 21 days for a total of eight cycles (combined regimen cohort). Treatment was planned to start within eight weeks after curative intent resection. Adverse events, disease-free survival and overall survival were assessed.

**Results:**

Overall 30 patients were enrolled in the study from August 2008 and last patient was enrolled at 2nd December 2014. The follow-up of the patients ended at 31st December 2016. The first 9 patients received single-agent gemcitabine. The interim analysis met the predefined feasibility criteria and, from September 2010 on, the second group of 21 patients received the combination of cisplatin plus gemcitabine. In the single-agent cohort with gemcitabine the median relative dose intensity (RDI) was 100% (IQR 88.3–100). Patients treated with the combination cisplatin-gemcitabine received an overall median RDI of 100% (IQR 50–100) for cisplatin and 100% (IQR 75–100) for gemcitabine respectively. The most significant non-hematological adverse events (grade 3 or 4) were fatigue (20%), infections during neutropenia (10%), and two cases of biliary sepsis (7%). Abnormal liver function was seen in 10% of the patients. One patient died due to infectious complications during treatment with cisplatin and gemcitabine. The median disease-free survival (DFS) was 14.9 months (95% CI 0–33.8) with a corresponding 3-year DFS of 43.1 ± 9.1%. The median overall survival (OS) was 40.6 months (95% CI 18.8–62.3) with a 3-year OS of 55.7 ± 9.2%. No statistically significant differences in survival were seen between the two treatment cohorts.

**Conclusion:**

Adjuvant chemotherapy with gemcitabine with or without cisplatin was well tolerated and resulted in promising survival of the patients.

**Trial registration:**

The study was retrospectively registered on 25th June 2009 at clinicaltrials.gov (NCT01073839).

## Background

Biliary tract cancer (BTC) arises from the biliary epithelium of intra- and extrahepatic bile ducts and the gallbladder. The incidence of intrahepatic cholangiocarcinomas has increased steadily in the last years with rising mortality rates, whereas the incidence of extrahepatic cholangiocarcinomas remained stable [1, 2].

Surgery is the only curative treatment for BTC patients, but only a minority of patients are cured [3]. Patients with cholangiocarcinoma have five-year survival rates of up to 20% for proximal lesions and 20–30% for distal lesions [4]. The prognosis for patients with gallbladder cancer is also unfavourable with overall five-year survival rates of less than 5%, with outcome depending largely on the stage of the disease at diagnosis [5]. The unfavourable prognosis of BTC provides the rationale to identify effective adjuvant treatment strategies for this disease. Two adjuvant phase III trials with different chemotherapy regimens, including single-agent gemcitabine, had been reported in patients with either resected gallbladder cancer or ampullary cancer, but they were not able to demonstrate a clear survival advantage [6–8]. One meta-analysis [9] evaluating the impact of adjuvant treatment with systemic chemotherapy, radiation, or combined chemoradiation in patients after BTC resection suggested a benefit for high-risk patients with positive lymph nodes or positive resection margins [10].

Single-agent chemotherapy with gemcitabine [11] has been the standard of care in the treatment of inoperable adenocarcinoma of the pancreas prior to more efficacious regimens such as FOLFIRINOX and gemcitabine plus nab-paclitaxel. Oettle and colleagues have shown that adjuvant chemotherapy with gemcitabine after macroscopic complete resection of pancreatic cancer prolongs disease free and overall survival compared to observation alone [12]. Since the biliary tract is being considered histologically related to the pancreatic duct system, gemcitabine has often been used as monotherapy in various smaller trials for the treatment of inoperable BTC with promising results [13–15].

A few years ago, new data for inoperable BTC became available [16]. In a randomized controlled phase 3 trial (ABC-02) cisplatin plus gemcitabine was compared to gemcitabine alone for locally advanced or metastatic biliary tract cancer. The median overall survival could be prolonged for almost four months from 8.1 months to 11.7 months with the combination regimen. In addition, the rate of tumor control among patients in the cisplatin-gemcitabine group was significantly increased (81.4% vs. 71.8%, *p* = 0.049) [16]. Both regimens were feasible with similar rates of adverse events in both groups, with the exception of increased neutropenia in the combination arm, which was not associated with an increased rate of infections. Based on this study, cisplatin plus gemcitabine is being considered the new practice standard for patients with inoperable biliary tract cancer.

We conducted this prospective, non-randomized study for patients undergoing macroscopic complete resection of BTC. Patients were initially treated with gemcitabine alone, and, after publication of the previous mentioned ABC-02 trial [13], we amended the protocol and added cisplatin to gemcitabine. We aimed to assess the feasibility of this adjuvant treatment in resected patients and its efficacy in terms of overall and disease free survival.

## Methods

**Fig. 1:**
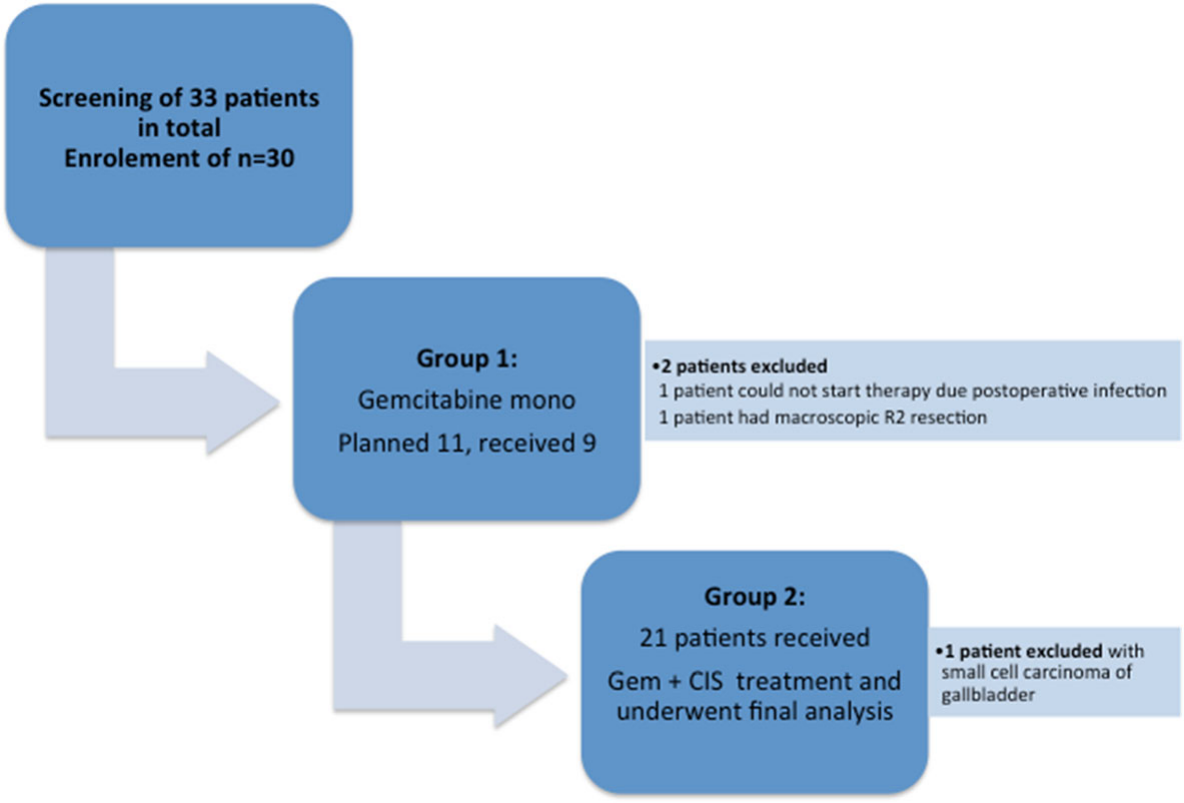
Enrollment, treatment group 1 and group 2

This trial was a prospective, single-arm phase II study conducted at the University Hospital Zurich in Switzerland. At the time of study conduct, no standard adjuvant treatment was yet established for cholangiocarcinoma, but gemcitabine had been established as adjuvant standard of care for resected pancreatic cancer shortly before by Oettle and colleagues. Based on these data and some smaller and retrospective data describing a benefit for gemcitabine in advanced cholangiocarcinoma, we chose this single agent treatment initially. The primary endpoints were safety and feasibility of adjuvant chemotherapy with the first patient cohort receiving gemcitabine as single-agent treatment and, after protocol amendment, the second patient cohort receiving the combination of cisplatin plus gemcitabine. After treatment of the first 11 patients, an interim safety analysis was planned, and the trial would have been stopped prematurely based on safety evaluations. Secondary endpoints were completion of treatment, adverse events according to CTCAE version 3.0, disease-free survival (DFS), and overall survival (OS). In addition, a separate analysis of the outcome in patients treated with gemcitabine alone (single agent cohort) and patients treated with cisplatin and gemcitabine combined (combined regimen cohort) was performed (Fig. 1). The trial started during August 2008 with first resection of BTC and ended by the cut-off date of patient’s follow up at 31st December 2016.

### Ethics approval and consent to participate

The study was conducted after obtaining approval from the local ethical committee (ethical number KEK Zurich 1442) at 15th January 2008 and Swissmedic by 2nd April 2008. First patient was included at 4th August 2008. Written informed consent was obtained from all patients in accordance with the Declaration of Helsinki. The study was retrospectively registered on 25th June 2009 at clinicaltrials.gov, as it was not yet obligatory at the time of trial start according to national specifications (NCT01073839).

### Eligibility criteria

Patients diagnosed with cholangiocellular carcinoma who underwent curative intent tumor resection were enrolled in this study at the Department of Oncology, University Hospital Zurich. The main eligibility criteria included the following: Histologically or cytologically confirmed adenocarcinoma of the biliary tract (intrahepatic, extrahepatic, gallbladder); resection of the tumor with curative intention up to 8 weeks before start of chemotherapy; written informed consent; health status: WHO performance status (PS) 0–1; age > 18 years; adequate renal function (creatinine clearance ≥60 ml/min, calculated according to the formula of Cockroft-Gault); adequate hepatic function (bilirubin ≤3 x LUN, AP ≤ 5 x LUN, ASAT ≤5 x LUN); adequate hematologic function: neutrophils ≥1.5 × 109/l, platelets ≥100 × 109/l, Hb ≥ 9,5 mg/dl.

Patients were excluded in case of: Pregnancy or breastfeeding women, previous malignancy within 5 years or concomitant malignancy except non-melanomatous skin cancer or adequately treated in situ cervical cancer, neutrophil count <1000/μl, platelet count <100,000/μl, hemoglobin level < 9,5 mg/dl, bilirubin >3 x LUN, ALAT >5 x LUN, ASAT >5 x LUN, creatinine clearance <60 ml/min, calculated according to the formula of Cockroft-Gault, prior chemotherapy with gemcitabine, severe or uncontrolled cardiovascular disease (congestive heart failure NYHA III or IV, unstable angina pectoris, history of myocardial infarction in the last 3 months, significant arrhythmias), psychiatric disorder precluding understanding of information of trial related topics and giving informed consent, active uncontrolled infection, pre-existing peripheral neuropathy (> grade 1), serious underlying medical condition (judged by the investigator) which could impair the ability of the patient to participate in the trial (e.g. uncontrolled diabetes mellitus, active autoimmune disease), concurrent treatment with other experimental drugs or other anti-cancer therapy; treatment in a clinical trial within 30 days prior to trial entry, known hypersensitivity to the study drug.

### Treatment schedule

According to the initial protocol version, the first patients enrolled received gemcitabine at a dose of 1000 mg/m^2^ as a 30-min infusion on days 1, 8 and 15 every 28 days for a total of 6 cycles (24 weeks in total). After treatment of 11 patients, the first interim analysis was performed in September 2010, and the study would continue with inclusion of additional 19 patients if feasibility could be shown. After this planned interim analysis, the protocol was amended on the evidence of new available data for the combination therapy with cisplatin and gemcitabine. In detail, the second cohort of patients received gemcitabine at 1000 mg/m^2^ and cisplatin at 25 mg/m^2^ on days 1 and 8 every 21 days for a total of 8 cycles (24 weeks of treatment in total). The treatment was stopped ahead of schedule in case of unacceptable toxicities, tumor recurrence, or patient wish. If the administration of the planned chemotherapy was delayed for more than a month, the treatment was discontinued and the patient clinically followed thereafter. Blood examinations were performed on each day of treatment. To continue treatment as planned, a neutrophil count greater than 1000/μl, and a platelet count greater than 100,000/μl was required for each full dose of gemcitabine and cisplatin. Dose reductions were indicated at days 8 (combined regimen cohort) or 15 (single agent cohort) of each cycle in case of thrombocytopenia with platelets between 75,000 and 100,000/μl and/or absolute neutrophil counts between 500 and 1000/μl. The treatment was withheld if neutrophil count was below 500/μl or platelet count was below 75,000/μl. Treatment was restarted following hematologic recovery (neutrophil >1000/μl and/or platelets >100,000/μl, respectively. Administration of G-CSF was allowed, but discontinuation was required at least 2 days prior to the next administration of chemotherapy.

After end of treatment clinical visits and laboratory analyses were carried out every three months for the first two years, thereafter every 6 months for the next three years and thereafter at the discretion of the attending physician. Assessments with CT-scans of thorax and abdomen and/or MRI of the abdomen were performed after end of treatment and 6 months later, and thereafter at the discretion of the attending physician.

### Statistical analysis

Patient recruitment followed a Simon’s two-stage design; a maximum of anticipated non-laboratory adverse events ≥ grade 3 in up to 45% of patients was considered acceptable, whereas non-laboratory adverse events ≥ grade 3 in more than 70% were considered unacceptable, resulting in the regimen being considered not feasible. Eleven patients had been planned to be recruited in the first phase; and if 4 or fewer patients experience at least one Grade 3 or 4 non-laboratory toxicity, a further 19 patients would be recruited for a total of 30 evaluable patients. This study had 80% power to discriminate between these two levels at the 5% level of significance.

The relative dose intensity (RDI) was calculated as the ratio of the actual dose given during the study to the planned dose in the protocol. DFS would be calculated from the day of resection until locoregional recurrence, the development of distant metastases, second primary cancer, death from the same or other cancer, or treatment-related death. Overall survival was calculated from the day of resection until death. Follow-up is also being reported from the day of surgery until the final cut-off date at 31st December 2016. Patients alive would be censored at that time point. Continuous and ordinal variables were presented as median with interquartile range (IQR). DFS and OS were determined by the Kaplan-Meier method and Cox-regression. Median time to event was reported with 95% confidence interval (CI). Estimates at 3 years were presented with standard error. Hazard ratios (HR) of gemcitabine plus cisplatin vs. gemcitabine monotherapy were presented with 95% CI. A post hoc comparison of the two patient groups receiving either gemcitabine alone or combination therapy with cisplatin and gemcitabine was performed by the log-rank test.

## Results

### Patient characteristics

From August 2008 to December 2014, 33 patients who underwent resection for BTC were screened for inclusion into this study, and 30 patients were finally enrolled. Reasons for non-enrolment were diagnosis of small cell carcinoma of the gallbladder, postoperative infectious complications and early relapse of disease, and early start of palliative chemotherapy due to a macroscopic R2 resection. Of the five patients with gallbladder cancer, one had an incidental finding of cancer after cholecystectomy and underwent a completion oncological resection. The demographic and clinical characteristics of the 30 patients are shown in Table 1. Major postoperative complications were observed in four patients: a pancreatic fistula in one patient, a liver abscess in one patients, and two patients developed a bilioma, with one of these patients undergoing a bilioenteral neostomy and percutaneous transhepatic cholangiography and drainage (PTCD). All patients recovered by the time of starting chemotherapy. Treatment was initiated after a median of 50 days (IQR 40–65 days) after curative intent surgery.

**Table 1:** Patient characteristics (*n* = 30)

Characteristic	No. (%)
Age	Median (IQR)
	55.5 (51–65.5)
Gender	
Male	14 (47)
Female	16 (53)
ECOG Performance Status	
ECOG PS 0	21 (70)
ECOG PS 1	9 (30)
Tumor localization	
Extrahepatic	6 (20)
Intrahepatic	17 (57)
Gallbladder	5 (17)
Ampulla vateri	2 (7)
Stage TNM	
T1/T2/T3/T4	4 (13)/15 (50)/11 (37)/0 (0)
N0/1	20 (67)/10 (33)
Margin Status	
R0	28 (93)
R1	2 (7)
Operative Procedure^a^	
Major hepatectomy	17 (57)
Hepatopancreaticoduodenectomy	1 (3)
Gall bladder bed resection	9 (30)
Pancreaticoduodenectomy	5 (17)
Time of initiation of Chemotherapy (days)	Median (IQR) 49.5 (39.8–64.5)

Abbreviations: *IQR* Interquartile Range, *ECOG* Eastern Cooperative Oncology Group, *TNM* tumor node metastasis, *R* residual tumor after treatment

^a^Includes multiple counts

### Feasibility

After treatment of 11 patients the planned interim safety analysis was performed. Two patients in this group had to be excluded from analysis, as one patient could not start chemotherapy due to postoperative infectious complications and early relapse of disease. The second patient had a macroscopic R2 resection. Overall, 9 patients were analysed. Treatment was well tolerated. However non-hematologic adverse events of grade 3 and 4 developed during treatment in 3 patients (33%). Two patients (22%) developed hypertension grade 3, which was controlled with antihypertensive medication, and one patient had a self-limiting episode of dyspnea grade 3 (11%), explained by concomitant anemia (grade 3). After recovery of the hemoglobin levels the dyspnea resolved. As all these non-hematologic adverse events were self-limiting or medically controlled without the need for hospitalization, we considered the treatment feasible and safe. As the results of the ABCstudy [16] had been reported at that time, demonstrating similar rates of adverse events for gemcitabine alone and the combination of gemcitabine and cisplatin, we considered adding cisplatin to the gemcitabine treatment safe and completed recruitment with the combination regimen after protocol amendment. Thus, the study was continued at September 2010, and 22 additional patients were enrolled. One of the subsequently enrolled patients was diagnosed with small cell cancer of the gallbladder and was subsequently excluded from the analysis.

The median time to onset of chemotherapy after surgery was 48 days (IQR 38.5–56.5) in the gemcitabine monotherapy group, and 54 days (IQR 39.5–70.5) in the cisplatin-gemcitabine group, respectively. Completion rates were 98% among the gemcitabine monotherapy treated patients, and 81% for cisplatin and 87% for gemcitabine in the combination treatment group (Table 2). The median RDI of gemcitabine delivered in the monotherapy group was 100% (IQR 91–100).

**Table 2:** Comparison of treatments in the gemcitabine-only and cisplatin-gemcitabine group

	Group 1 (*n* = 9, GEM)	Group 2 (*n* = 21, CIS/GEM)
Time of initiation days; median (IQR)	48 (38.5–56.5)	54 (39.5–70.5)
Number of courses; median	6	8/8
Completion rate; %	98%	81%/88%
Dose reduction; no.	7	15

Abbreviations: *GEM* gemcitabine, *CIS* cisplatin, *IQR* interquartile range

Patients with the combination of cisplatin-gemcitabine showed a median dose intensity of 100% (IQR 50–100) for cisplatin and 100% (IQR 75–100) for gemcitabine (Table 3).

**Table 3:** Relative dose intensity (RDI) for the two treatment regimens

Dose Intensity Rates (DSI) in percentage							
GEM Group (*n* = 9)			CIS/GEM Group (*n* = 21)				
				CIS		GEM	
Cycles	Median	IQR	Cycles	Median	IQR	Median	IQR
C1	100	(91.7–100)	C1	100	(100–100)	100	(100–100)
C2	100	(95–100)	C2	100	(100–100)	100	(100–100)
C3	100	(90.9–100)	C3	100	(70–100)	100	(76.4–100)
C4	100	(86.7–100)	C4	80	(50–100)	100	(77.5–100)
C5	100	(95–100)	C5	80	(50–100)	100	(70–100)
C6	100	(88.9–100)	C6	80	(25–100)	94.1	(70–100)
			C7	50	(0–100)	88.9	(56.3–100)
			C8	50	(0–90)	75	(50–100)
Overall DSI	100	(91–100)		100	(50–100)	100	(75–100)

Abbreviations: *GEM* gemcitabine, *CIS* cisplatin, *IQR* interquartile range, *C* cycles

In the gemcitabine monotherapy group, treatment was discontinued in one patient because of not further specified recurrent abdominal infections and pulmonary fistula. In the cisplatin-gemcitabine combination group treatment was discontinued ahead of schedule in 9 patients due to following reasons: neuropathy (*n* = 1), early tumor recurrence (*n* = 1), fatigue (*n* = 2), renal impairment (*n* = 2), septic shock and death (*n* = 1), and hematologic adverse events (*n* = 2).

### Adverse events

Hematologic and non-hematologic adverse events of grade 3 and 4 are listed in Table 4. The main adverse events were hematologic toxicities with leukopenia (27%) and neutropenia (50%). Anemia was documented in 10% and thrombocytopenia in 17% in total for both groups. Mentionable non-hematologic toxicities of grade 3 and 4 were fatigue in 6 (20%) patients and infections in three (10%) patients. Infections were only seen in the cisplatin-gemcitabine group. Two patients (7%) receiving the combination treatment developed biliary sepsis. Renal dysfunction was observed in two patients (7%) receiving the combination. One treatment-related death was observed in a patient who developed a septic shock due to a pulmonary infection during the second cycle of cisplatin and gemcitabine. Overall, non-hematological adverse events grade 3 or greater related to chemotherapy were observed in 11 (37%) patients during treatment, in three (33%) patients receiving gemcitabine alone and in eight (38%) patients receiving cisplatin plus gemcitabine.

**Table 4:** Grade 3 and 4 adverse events. Reported is the highest grade observed

Events^a^	Grade 3 - 4^b^ GEM (*n* = 9)	Grade 3 - 4^b^ CIS/GEM (*n* = 21)	Total
	No. (%)		
Hematologic toxic effects			
Leukopenia	0	8 (38)	8 (27)
Neutropenia	3 (33)	12 (57)	15 (50)
Thrombocytopenia	1 (11)	4 (19)	5 (17)
Anaemia	0	3 (14)	3 (10)
Non-hematologic toxic effects			
Alopecia	0	2 (10)	2 (7)
Anorexia	0	2 (10)	2 (7)
Fatigue	3 (33)	4 (19)	6 (20)
Nausea	0	1 (5)	1 (3)
Vomiting	0	1 (5)	1 (3)
Impaired renal function	0	2 (5)	2 (7)
Infection	0 (0)	3 (14)	3 (10)
Without neutropenia	0	0	0 (0)
With neutropenia	0	3	3 (10)
Biliary sepsis	0	2	2 (7)
Deep-vein thrombosis	0	1 (5)	1 (3)
Other	1 (11)	5 (24)	6 (20)
Liver function			
Increased ALAT level	0	1 (5)	1 (3)
Other abnormal liver function^c^	0	2 (10)	2 (7)
Any abnormal liver function^d^	0	0 (0)	0 (0)

Abbreviations: *GEM* gemcitabine, *CIS* cisplatin, *ALAT* alanine aminotransferase

^a^Multiple adverse events per patient possible

^b^Pre-existing conditions are reported only in case of worsening during study treatment

^c^Elevated gamma-GT

^d^Hypoalbumia, decreased Vitamin K level

### Survival

**Fig. 2:**
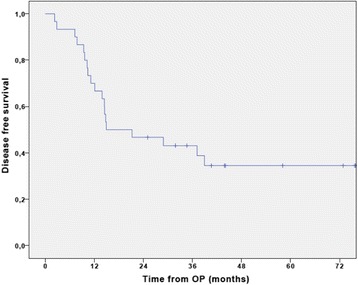
Cumulative disease free survival curve of the study population (*n* = 30). The median DFS was 14.9 months (95% CI 0–33.8) for the entire patient population with a 3-year DFS of 43.1 ± 9.1%

**Fig. 3:**
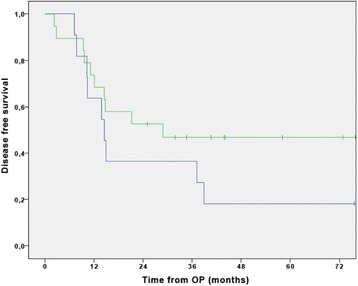
Disease free survival of gemcitabine-mono (blue curve) and cisplatin-gemcitabine (green curve) patients. The median DFS of the patients receiving gemcitabine plus cisplatin was 28.8 months (95% CI not available), and 14.4 months (95% CI 9.5–19.3) in the patients receiving gemcitabine alone

The median follow-up time was 31.4 months (IQR, 23.2–49.5 months) at the time of data cut-off. Eleven patients were still alive at this time point. The median DFS was 14.9 months (95% CI 0–33.8) for the entire patient population with a 3-year DFS of 43.1 ± 9.1%. (Fig. 2). The median DFS of the patients receiving gemcitabine plus cisplatin was 28.8 months (95% CI not available), and 14.4 months (95% CI 9.5–19.3) in the patients receiving gemcitabine alone (Fig. 3). No differences were seen in an exploratory post hoc comparison (HR 0.57 (95% CI 0.23–1.4); *p* = 0.22) between the two treatment groups.

**Fig. 4:**
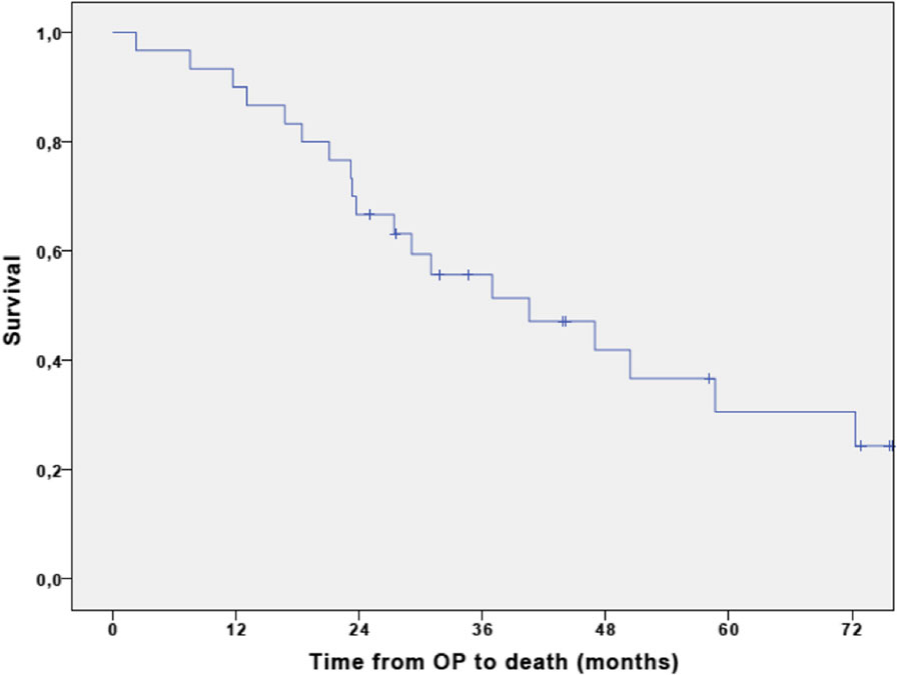
Cumulative overall survival curve of the study population (*n* = 30). The median overall survival of the whole patient collective was 40.6 month (95% CI 18.8–62.3) with a 3-year OS of 55.7 ± 9.2%

**Fig. 5:**
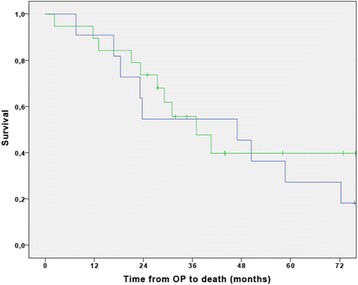
Overall survival of gemcitabine-mono (blue curve) and cisplatin- gemcitabine (green curve) patients. Patients receiving cisplatin plus gemcitabine had a median OS of 36.9 months (95% CI 22.1–51.7), and patients receiving gemcitabine alone had a median survival of 46.9 months (95% CI 17.5–76.3)

The median overall survival of the whole patient collective was 40.6 month (95% CI 18.8–62.3) with a 3-year OS of 55.7 ± 9.2% (Fig. 4). Patients receiving cisplatin plus gemcitabine had a median OS of 36.9 months (95% CI 22.1–51.7), and patients receiving gemcitabine alone had a median survival of 46.9 months (95% CI 17.5–76.3) (Fig. 5). No statistically significant difference was seen between the two groups in a post hoc comparison (HR 0.82 (95% CI 0.32–2.1); *p* = 0.67).

### Treatment after tumor recurrence

Overall, 18 tumor recurrences were observed until end of follow-up. Local, distant, and combined local and distant recurrences were seen in 9, 3, and 6 patients, respectively. Patients initially treated with gemcitabine alone were offered the combination of cisplatin plus gemcitabine at the time of recurrence. Patients who suffered tumor recurrence during or after treatment within the cisplatin-gemcitabine cohort were offered various treatment regimens, chosen according to the performance status of the patients. Treatment consisted of FOLFIRINOX, FOLFOX, FOLFIRI or, in later lines, cisplatin-etoposide or carboplatin-etoposide. One patient received cetuximab combined with fluorouracil, oxaliplatin and leucovorine. Three patients with local recurrences received selective intraarterial radiotherapy (SIRT). One patient received radiochemotherapy with capecitabine. No patient was able to undergo secondary resection.

## Discussion

This prospective phase II study was able to document the feasibility of adjuvant chemotherapy with gemcitabine alone or combined with cisplatin in patients after curative intent surgery for BTC. Assessment of safety was our primary objective, as patients frequently have to cope with postoperative complications like fatigue, hepatobiliary infections and impaired renal or liver function after major liver resection. The number of patients developing higher grades of non-hematological adverse events during treatment did not exceed the predefined cut-off, and adjuvant chemotherapy was thus deemed safe. With a median follow up time of 31 months we were able to record a 3-year DFS of 43% and a 3-year OS of 56% in our patient population. These data are well in line with recently published retrospective analyses, which suggest a survival advantage from adjuvant chemotherapy [17–21].

Our data highlight that single agent gemcitabine and the combination of cisplatin plus gemcitabine are both feasible options for adjuvant treatment of patients who had undergone macroscopic complete resection of biliary tract cancer. Hematologic adverse events like neutropenia were more frequently noted in the combination group than in the gemcitabine monotherapy group (63% vs 27%), and, accordingly, also higher rates of infections were seen with the combination treatment (16% vs. 9%). One explanation for this finding may be that the preceding hepatobiliary resection may result in subsequent postoperative complications like fistulation or biliomas, which predispose the patients toward infectious complications per se. This high rate of infectious complications in the combination regimen cohort highlights that patient recovery after surgery is of utmost importance and needs to be achieved before potentially hematotoxic chemotherapy can be administered. Despite the fact that we were not able to start adjuvant treatment in all patients within the anticipated eight-week interval after resection due to delayed recovery time, the RDI was rather high in both patient cohorts, underscoring the feasibility of adjuvant chemotherapy in this patient population. Median RDI of 100% could be reached with gemcitabine alone and the cisplatin-gemcitabine combination as well, demonstrating very good tolerability of both regimens in the post-operative adjuvant setting.

The optimal adjuvant management of patients undergoing curative intent resection of BTC has not been defined as of completion of this study. Several practice guidelines, i.e. by ESMO and NCCN, are in place, which recommend the use of adjuvant chemotherapy with platinum based chemotherapy or chemoradiation in the adjuvant setting within clinical trials [22]. However, these recommendations are based predominantly on retrospective data, small series or personal practice.

Only few prospective trials studying the role of adjuvant treatment combining gemcitabine with another chemotherapeutic partner are available [23–25]. They evaluated the feasibility of these regimens in predominantly Asian patient populations and reported similar survival outcome as in the present study.

A few randomized phase III trials are currently being conducted and are assessing the impact of different chemotherapy regimens after macroscopically complete resection of BTC [26, 27]. One phase III study evaluating gemcitabine in combination with oxaliplatin (PRODIGE 12) showed that the combination chemotherapy was feasible, but found no difference in relapse free survival compared to the observation group [28]. A second phase III study (BILCAP) testing single agent capecitabine in the adjuvant setting was recently reported and showed, in contrast to the aforementioned PRODIGE 12, a signal in favour of adjuvant chemotherapy [29]. Although the study failed to demonstrate a significant improvement of overall survival in the intention to treat analysis, the median survival was markedly longer if patients were treated with capecitabine as per protocol [30]. The better outcome may be attributed to a higher number of patients with nodal-positive disease included in the latter study compared to the PRODIGE 12. This explanation is being supported by a statistically significant difference in overall survival after adjusting for various risk factors, including nodal status, in a prespecified sensitivity analysis of the BILCAP study, suggesting that these patients may derive the most benefit from adjuvant chemotherapy. Based on these results, adjuvant capecitabine may evolve into a new practice standard after curative resection of BTC. Finally, a large multi-national study (ACTICCA-1) is evaluating the combination of gemcitabine plus cisplatin with results being expected in the next years. As our study was planned and set up before this phase III trial had been initiated, we are able to provide valuable information on the feasibility and efficacy of adjuvant gemcitabine and cisplatin in patients with resected cholangiocarcinoma at a time when no other data are available for this doublet. As extrahepatic BTC may have a different biological behaviour than intrahepatic cancers, a subgroup analysis would be desirable to evaluate which patients may derive the most benefit of this doublet regimen. If we look at the large ABC-02 trial, the addition of cisplatin to gemcitabine resulted in a comparable advantage in terms of survival for all subgroups of patients, irrespective of the origin of the biliary tumor, suggesting that all patients with BTC may achieve a benefit from this combination. The results of the ACTICCA-1 study and further analyses of the BILCAP study will hopefully identify subsets of patients who may benefit in particular from a specific regimen.

Our study has some limitations. First, due to the low number and the heterogeneity of patients included, this study was not sufficiently powered to allow meaningful subgroup analyses, for instance with regard to nodal or resection margin positivity and the aforementioned tumor localization. Our post hoc comparison of the two treatment groups is therefore only exploratory. Second, the study was planned and conducted at a single institution in Switzerland. As cholangiocarcinoma is still to be considered a rather rare disease compared to pancreatic cancer, patient availability for enrolment into this study was too low to allow for a rapid completion within a few years. In addition, the study had to be amended to adapt the treatment regimen, and halting the patient accrual for some months was necessary, which further delayed the completion of the study. Being confronted with these barriers, we were able to establish a patient referral system with various regional centers for this specific study, which improved accrual rates and helped to finish the study.

## Conclusions

In conclusion, our study showed that treatment with gemcitabine either alone or combined with cisplatin is feasible and well tolerated in patients with curative resected biliary tract cancer. Toxicities were within the expected range, and survival rates were promising. The multi-national prospective phase III trial ACTICCA-1 addressing the question of adjuvant gemcitabine and cisplatin is currently underway and will help to identify the optimal regimen for this difficult to treat patient population.

## Abbreviations

ALAT: alanine aminotransferase
C: Cycles
CIS: Cisplatin
ECOG: Eastern Cooperative Oncology Group
GEM: Gemcitabine
HPD: Hepatectomy concomitant with pancreatoduodenectomy
IQR: Interquartile Range
R: Residual tumor after treatment
TNM: Tumor node metastasis
